# Video-assisted thoracic surgery for primary myelolipoma of the posterior mediastinum

**DOI:** 10.1186/s13019-016-0401-7

**Published:** 2016-01-13

**Authors:** Naoya Himuro, Takao Minakata, Yutaka Oshima, Yuri Tomita, Daisuke Kataoka, Shigeru Yamamoto, Mitsutaka Kadokura

**Affiliations:** Division of Chest Surgery, Department of Surgery, Showa University School of Medicine, 1-5-8 Hatanodai, Shinagawa-ku, Tokyo Japan; Division of Chest Surgery, Showa University School of Medicine, 1-5-8 Hatanodai, Shinagawa-ku, Tokyo 142-8666 Japan

**Keywords:** Posterior mediastinal tumor, Myelolipoma, Extramedullary hematopoiesis, Video-assisted thoracic surgery

## Abstract

**Background:**

Myelolipoma is an uncommon tumor comprising adipose tissue and normal hematopoietic cells and mainly occurs in the adrenal cortex. Mediastinal myelolipoma is very rare; we report a case of posterior mediastinal myelolipoma that required surgical resection.

**Case Presentation:**

A 56-year-old male was diagnosed with a posterior mediastinal tumor by computed tomography. The tumor was originally noted in 2005, and during follow-up in March 2014, it was found to have increased in size. During consultation at our hospital, on magnetic resonance imaging (MRI), we considered the possibility that the tumor was malignant. Consequently, we resected the tumor by video-assisted thoracic surgery (VATS). The histopathological findings revealed that the tumor had undergone intrathoracic extramedullary hematopoiesis. However, after considering the patient’s background and histopathological findings, we diagnosed the tumor as a thoracic extra-adrenal myelolipoma.

**Conclusions:**

Pathological analysis was instrumental in clarifying the diagnosis. We recommend surgery as a treatment option for posterior mediastinal tumors.

## Background

Primary mediastinal myelolipoma is an extremely rare benign tumor and should be considered in the differential diagnosis of posterior mediastinal tumors. It is difficult to distinguish myelolipoma and extramedullary hematopoiesis using only histopathological examination. Therefore, it is important to diagnose tumors by histopathological findings associated with clinical background. Here, we report a surgical case of mediastinal myelolipoma.

## Case Presentation

**Fig. 1:**
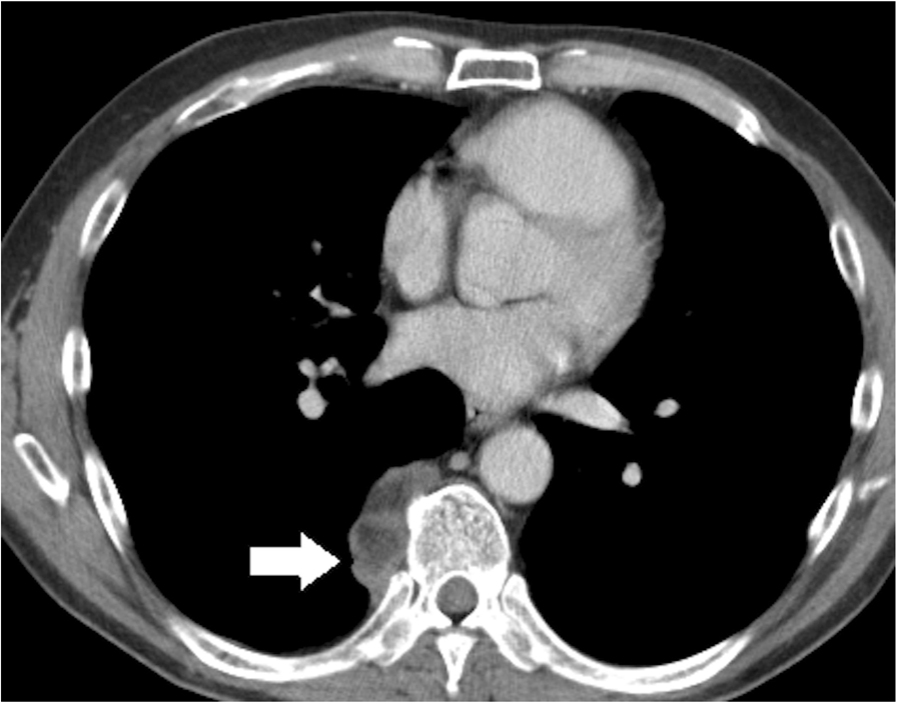
Chest CT at our hospital showed a tumor in the right posterior mediastinum (arrow). It measured 4.3 cm in diameter and was located beside the 9th thoracic vertebrae

**Fig. 2:**
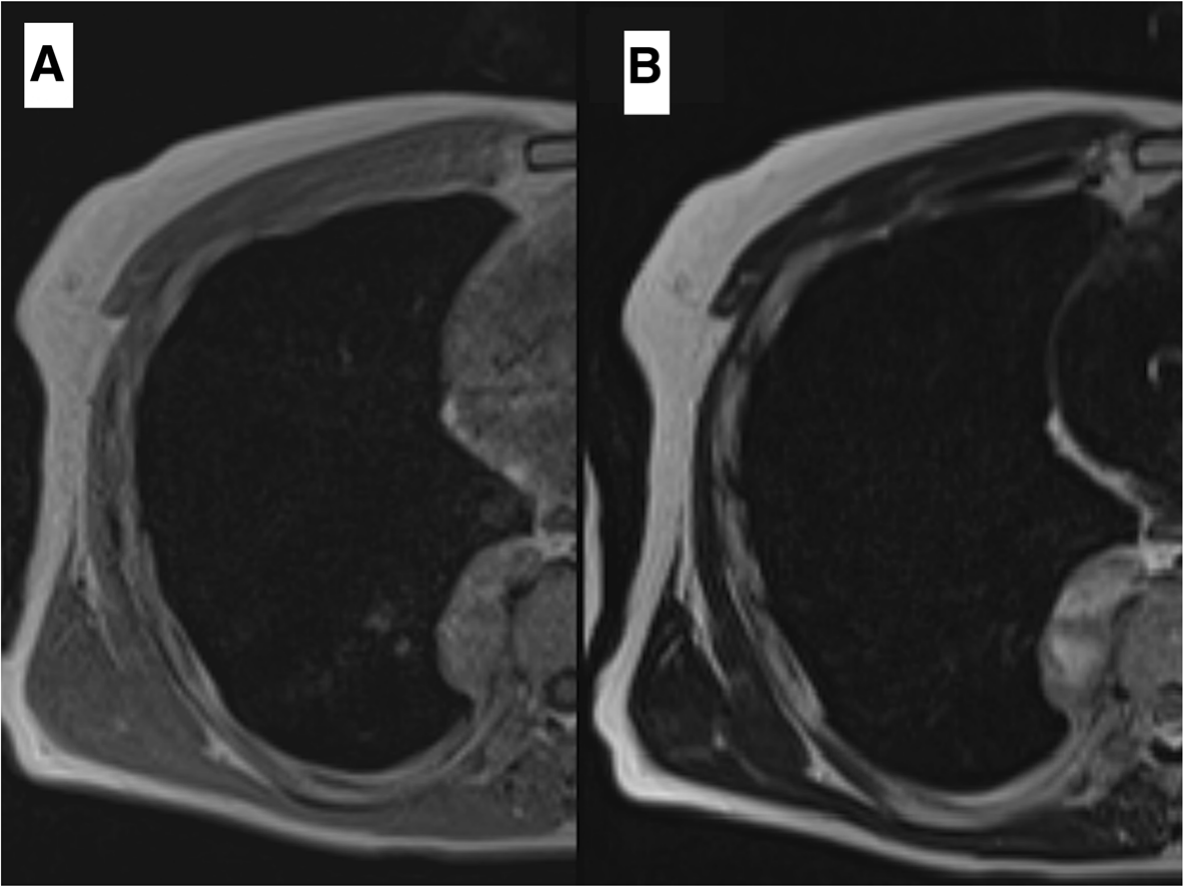
Chest MRI revealed that the signal intensity of the tumor was low in the muscles in the T1 weighted image (**a**) and disproportionately high in the T2 weighted image (**b**). The tumor invaded neither the intervertebral foramen nor the vertebral body

A 56-year-old male was referred to our hospital for surgical resection of a posterior mediastinal tumor in July 2014. A local doctor had originally diagnosed the tumor in 2005, and it had gradually increased in size until March 2014. The patient did not have any health problems or symptoms suggestive of a history of hematologic disorder or chronic anemia. Laboratory tests for tumor detection, including those implementing tumor markers, were within normal limits. A chest roentgenogram and computed tomography (CT) showed a tumor mass shadow in the right lower posterior mediastinum. The tumor was 4.3 cm in diameter and was evident on the side of the T9 thoracic vertebrae (Fig. 1). Magnetic resonance imaging (MRI) revealed that the signal intensity of the tumor was low in the T1 weighted image and disproportionately high in the T2 weighted image (Fig. 2).

**Fig. 3:**
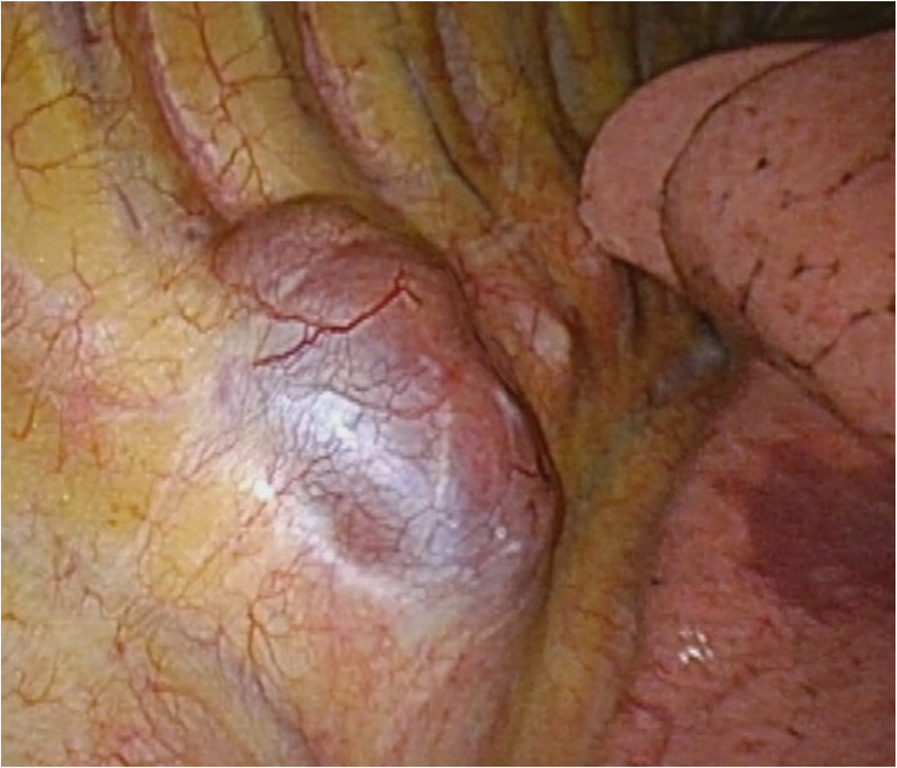
Operation findings identified a dark red, well-encapsulated, and elastic soft mass in the posterior mediastinum

Differential diagnoses included neurogenic tumor, malignant lymphoma, pleural mesothelioma, lipoma, liposarcoma, or an extra-adrenal myelolipoma. We performed tumor resection by video-assisted thoracic surgery (VATS). Surgery revealed that the tumor in the posterior mediastinum was a well-encapsulated, elastic, soft, and dark red mass (Fig. 3).

**Fig. 4:**
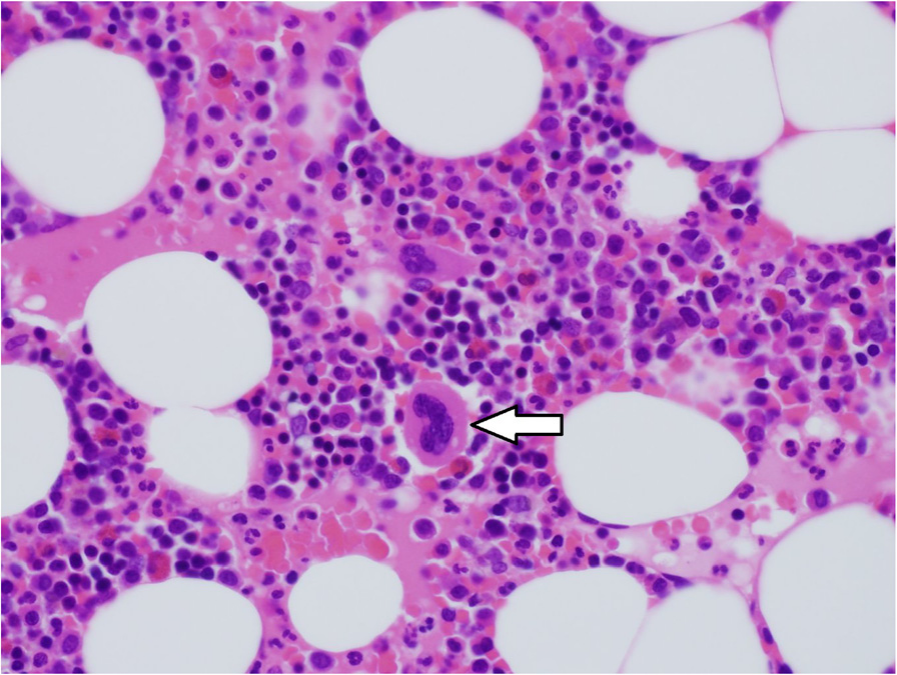
Microscopic examination showed adipocytes mixed with hematopoietic elements, including megakaryocytes (arrow) and regular granulopoietic and erythropoietin lineage cells

Histopathological findings of the resected specimens revealed a predominantly mature adipose tissue with hematopoietic tissue comprised of erythroblasts, megakaryocytes, and granulocytes (Fig. 4).

Although extramedullary hematopoiesis was considered as a differential diagnosis, the final diagnosis was a mediastinal extra-adrenal myelolipoma. The patient was discharged with no complications on the fifth postoperative day, and he has been well for 12 months postoperatively.

## Discussion

Gierke first described Myelolipoma in 1905, and the term “myelolipoma” was also coined by Oberling in 1929 [1–3]. Myelolipoma is a benign, nonfunctional tumor comprising extensive hematopoietic tissue and sparse fatty tissue. Most cases are hormonally inactive and found incidentally. The extra-adrenal location of myelolipoma is extremely unusual, and these tumors have been reported in the retroperitoneum, stomach, liver, mediastinum, bilateral paravertebral sulci [4], lung [5], presacral and perirenal spaces, and thoracic spine [6].

Chest CT and MRI are useful in clinical diagnosis. CT shows the mass shadow with a smooth, clear border and a localized low-density area. However, MRI shows high-intensity regions on both T1 and T2-weighted images. These findings are helpful to reach a definitive diagnosis [3, 7].

Myelolipomas located in the mediastinum are usually asymptomatic, and there have been no reports of myelolipomas transforming into malignant tumors. The decision-making process for surgical resection is based on a number of factors, including increased tumor size or local symptoms, such as chest pain, hemothorax, or paralysis, caused by spinal cord compression [3, 8]. We considered neurogenic tumor, malignant lymphoma, liposarcoma, and pleural mesothelioma in the differential diagnosis but conclusive evidence for these diagnoses was lacking. A CT-guided fine needle biopsy has been reported useful to avoid surgery. However, the use of needle biopsy can augment the risk of bleeding and tumor rupture [9, 10]. The differential diagnosis of thoracic extra-adrenal myelolipoma and thoracic extramedullary hematopoiesis cannot be confirmed purely on a pathological basis as both these conditions feature hematopoietic elements and adipose tissue [11]. Although thoracic extramedullary hematopoiesis occurs at multiple sites as lobulated tumors in the thoracic paravertebral area, thoracic extra-adrenal myelolipoma predominantly occurs at a single site as an encapsulated tumor [8].

Patients with extramedullary hematopoiesis usually have hematological disorders, such as anemia, leukemia, and myelodysplastic syndrome. However, extra-adrenal myelolipomas are not typically associated with anemia or other hematological disorders. From the histopathological findings and the clinical background of the patient, we diagnosed the tumor to be an extra-adrenal myelolipoma. Histopathological findings are effective elements in clarifying the diagnosis. There is no standard treatment for primary mediastinal myelolipoma; however, if such tumors start to increase in size or cause symptoms, they should be surgically resected. Due to the progressive enlargement of the tumor and the uncertain pre-operative diagnosis of this case, we removed the tumor by VATS. No recurrence or malignant transformation of primary mediastinal myelolipomas has been reported, so the long-term prognosis is very good.

## Conclusions

We experienced a case of myelolipoma in the mediastinum. Although primary mediastinal myelolipoma is very rare, knowledge of this tumor is important for correct differential diagnosis of the mediastinal tumor.

### Consent

Written informed consent was obtained from the patient for the publication of this case report and any accompanying images.

## Abbreviations

CT: Computed tomography
MRI: Magnetic resonance imaging
VATS: Video-assisted thoracic surgery
